# Supplementary material for: A Critical Issue in Lung Cancer Cytology and Small Biopsies: DNA and RNA Extraction from Archival Stained Slides for Biomarker Detection through Real Time PCR and NGS—The Experience in Pathological Anatomy Unit
Source: Diagnostics (Basel). 2023 May 5;13(9):1637. doi: 10.3390/diagnostics13091637 (PMC10178763; doi:10.3390/diagnostics13091637)
Supplement: Supplementary file 1 [file diagnostics-13-01637-s001.zip › Table S1.pdf]

Table S1. Years of collection of the samples analyzed in our series.

SAMPLES ANALYZED					
HISTOLOGICAL		SMALL BIOPSIES		CYTOLOGICAL	
**Cases**	**Years of Collection**	**Cases**	**Years of Collection**	**Cases**	**Years of Collection**
1	2019	1	2021	1	2019
2	2019	2	2020	2	2019
3	2021	3	2019	3	2019
4	2021	4	2019	4	2021
5	2020	5	2021	5	2020
6	2022	6	2019	6	2019
7	2019	7	2020	7	2022
8	2021	8	2020	8	2021
9	2023	9	2022	9	2019
10	2019	10	2022	10	2019
11	2020	11	2023	11	2023
12	2020	12	2021	12	2021
13	2019	13	2021	13	2021
14	2019	14	2020	14	2021
15	2022	15	2023	15	2023
16	2023			16	2020
17	2023			17	2019
18	2021			18	2019
19	2020			19	2019
20	2020			20	2023
21	2022			21	2022
22	2022			22	2021
23	2023			23	2021
24	2021			24	2020
25	2021			25	2020
26	2021			26	2023
27	2021			27	2023
28	2023			28	2019
29	2023			29	2019
30	2020			30	2021
31	2020			31	2020
32	2020			32	2023
33	2023			33	2023
34	2022			34	2022
35	2022			35	2019
				36	2021
				37	2020
				38	2020
