# Supplementary material for: A Critical Issue in Lung Cancer Cytology and Small Biopsies: DNA and RNA Extraction from Archival Stained Slides for Biomarker Detection through Real Time PCR and NGS—The Experience in Pathological Anatomy Unit
Source: Diagnostics (Basel). 2023 May 5;13(9):1637. doi: 10.3390/diagnostics13091637 (PMC10178763; doi:10.3390/diagnostics13091637)
Supplement: Supplementary file 1 [file diagnostics-13-01637-s001.zip › Table S2.pdf]

Table S2. Quality reports of NGS and Real Time PCR data.
(EGFR L858R mutation ΔCq cut-off: ≤ 10.0, according to EasyPGX® ready EGFR kit manufacturer's instructions).

MSSs ELIGIBLE FOR NGS DNA-BASED													
**Types of samples**	**Case ID**	**NGS ANALYSIS**							**REAL TIME PCR ANALYSIS**				**Concordance between NGS and Real Time PCR results**
									**EGFR**		**BRAF**		
		**Mean Coverage**	**Uniformity of Coverage (%)**	**Raw Reads**	**Valid Reads**	**On-target Reads (%)**	**EGFR**	**BRAF**	**ΔCq**	**Status**	**ΔCq**	**Status**	
**HISTOLOGICAL (n. 18)**	1	11939	81.69	1173200	1084787	99.99	WT	WT	No Cq	WT	No Cq	WT	Yes
	2	5941	74.72	636554	551818	99.95	WT	WT	No Cq	WT	No Cq	WT	Yes
	3	10781	76.43	1111858	995813	99.98	WT	WT	No Cq	WT	No Cq	WT	Yes
	4	7202	78.80	776006	671223	99.96	WT	WT	No Cq	WT	No Cq	WT	Yes
	5	6916	79.59	738252	628387	99.98	WT	WT	No Cq	WT	No Cq	WT	Yes
	7	5631	74.70	629558	530582	99.97	WT	WT	No Cq	WT	No Cq	WT	Yes
	9	5828	75.63	653056	543914	99.99	WT	WT	No Cq	WT	No Cq	WT	Yes
	10	6630	75.26	786558	617566	99.97	WT	WT	No Cq	WT	No Cq	WT	Yes
	11	6523	72.79	712930	600259	99.98	WT	WT	No Cq	WT	No Cq	WT	Yes
	12	5155	87.43	598048	473006	99.97	WT	WT	No Cq	WT	No Cq	WT	Yes
	14	4514	92.60	536818	413831	99.90	WT	WT	No Cq	WT	No Cq	WT	Yes
	18	5441	90.32	596268	502279	99.97	c.2573T>G (L858R) ex 21	WT	2.0	MUT	No Cq	WT	Yes
	19	4863	91.19	545340	452561	99.96	WT	WT	No Cq	WT	No Cq	WT	Yes
	28	7408	91.27	819144	680007	99.93	WT	WT	No Cq	WT	No Cq	WT	Yes
	30	7043	96.24	777478	649193	99.98	WT	WT	No Cq	WT	No Cq	WT	Yes
	32	6806	91.96	799466	627420	99.95	WT	WT	No Cq	WT	No Cq	WT	Yes
	33	7063	87.36	887742	647748	99.95	WT	WT	No Cq	WT	No Cq	WT	Yes
	35	6435	93.59	778258	591751	99.91	WT	WT	No Cq	WT	No Cq	WT	Yes
**SMALL BIOPSIES (n. 11)**	1	6091	96.05	693218	556776	99.87	WT	WT	No Cq	WT	No Cq	WT	Yes
	2	5617	82.89	660946	532491	99.98	WT	WT	No Cq	WT	No Cq	WT	Yes
	4	10734	78.59	1120470	985576	99.98	WT	WT	No Cq	WT	No Cq	WT	Yes
	7	5662	89.52	649946	520117	99.93	WT	WT	No Cq	WT	No Cq	WT	Yes
	8	12317	82.29	1287846	1143996	99.96	WT	WT	No Cq	WT	No Cq	WT	Yes
	9	4877	87.61	1141620	992098	99.93	WT	WT	No Cq	WT	No Cq	WT	Yes

	10	5086	85.26	1202308	1042434	99.88	WT	WT	No Cq	WT	No Cq	WT	Yes
	11	6217	87.18	1474906	1259014	99.93	WT	WT	No Cq	WT	No Cq	WT	Yes
	13	11454	61.89	3011618	2551070	99.68	WT	WT	No Cq	WT	No Cq	WT	Yes
	14	10383	72.72	1092934	961568	99.97	WT	WT	No Cq	WT	No Cq	WT	Yes
	15	9912	73.69	1020548	916144	99.97	WT	WT	No Cq	WT	No Cq	WT	Yes
**CYTOLOGICAL (n. 26)**	1	3603	71.12	700190	449133	99.98	WT	WT	No Cq	WT	No Cq	WT	Yes
	2	4155	73.31	769042	516138	99.99	WT	WT	No Cq	WT	No Cq	WT	Yes
	3	3437	69.38	846782	429421	99.97	WT	WT	No Cq	WT	No Cq	WT	Yes
	4	5065	69.55	759778	628526	99.95	WT	WT	No Cq	WT	No Cq	WT	Yes
	6	3151	69.53	419946	300127	98.81	WT	WT	No Cq	WT	No Cq	WT	Yes
	11	4966	92.96	590226	477608	99.98	WT	WT	No Cq	WT	No Cq	WT	Yes
	12	4139	84.40	1212546	968986	99.62	WT	WT	No Cq	WT	No Cq	WT	Yes
	13	4940	69.32	524222	483082	99.99	WT	WT	No Cq	WT	No Cq	WT	Yes
	14	4044	65.73	462494	429339	99.99	WT	WT	No Cq	WT	No Cq	WT	Yes
	15	3231	85.85	1508370	735938	99.99	WT	WT	No Cq	WT	No Cq	WT	Yes
	16	3702	84.61	1214370	816424	99.85	c.2573T>G (L858R) ex 21	WT	2.0	MUT	No Cq	WT	Yes
	17	3764	90.99	1116112	814700	99.99	WT	WT	No Cq	WT	No Cq	WT	Yes
	18	1095	89.29	321792	242184	99.88	WT	WT	No Cq	WT	No Cq	WT	Yes
	19	2739	90.45	763566	611836	99.90	WT	WT	No Cq	WT	No Cq	WT	Yes
	20	3576	74.03	390326	329894	99.96	WT	WT	No Cq	WT	No Cq	WT	Yes
	22	3280	78.0	339198	299554	99.96	WT	WT	No Cq	WT	No Cq	WT	Yes
	23	3677	78.2	403984	337955	99.98	WT	WT	No Cq	WT	No Cq	WT	Yes
	24	3929	76.09	425710	364116	99.98	WT	WT	No Cq	WT	No Cq	WT	Yes
	25	3355	76.96	370362	309575	99.98	WT	WT	No Cq	WT	No Cq	WT	Yes
	26	3299	76.38	361650	305629	99.96	WT	WT	No Cq	WT	No Cq	WT	Yes
	28	3389	72.41	394458	316867	99.97	WT	WT	No Cq	WT	No Cq	WT	Yes
	29	3321	75.71	374354	306918	99.97	WT	WT	No Cq	WT	No Cq	WT	Yes
	31	3582	74.17	406956	331254	99.95	WT	WT	No Cq	WT	No Cq	WT	Yes
	32	3372	78.21	357222	305658	99.95	WT	WT	No Cq	WT	No Cq	WT	Yes
	35	2790	82.22	629526	540746	99.95	WT	WT	No Cq	WT	No Cq	WT	Yes
	36	1377	77.95	568188	319692	99.83	WT	WT	No Cq	WT	No Cq	WT	Yes

MSSs: mixed stained slides; ex: exon; WT: wild type; MUT mutated.
